# Non-Antimicrobial Adjuvant Therapy Using Ticagrelor Reduced Biofilm-Related *Staphylococcus aureus* Prosthetic Joint Infection

**DOI:** 10.3389/fphar.2022.927783

**Published:** 2022-07-01

**Authors:** Narayan Pant, Socorro Miranda-Hernandez, Catherine Rush, Jeffrey Warner, Damon P. Eisen

**Affiliations:** ^1^ College of Medicine and Dentistry, James Cook University, Townsville, QLD, Australia; ^2^ Australian Institute of Tropical Health and Medicine, Townsville, QLD, Australia

**Keywords:** biofilm-related prosthetic joint infection, adjuvant therapy, *S. aureus*, ticagrelor, animal model

## Abstract

**Background:** Prosthetic joint infection (PJI), frequently caused by *Staphylococcus aureus*, leads to a significant arthroplasty failure rate. Biofilm is a crucial virulence factor of *S. aureus* that is intrinsic to the pathogenesis of PJI. Biofilm-related infections are recalcitrant to antibiotic treatment. Surgical and antibiotic therapy could be combined with non-antibacterial adjuvants to improve overall treatment success. Ticagrelor, a P2Y12 receptor inhibitor antiplatelet drug, is known to have anti-staphylococcal antibacterial and antibiofilm activity. However, the molecular mechanism for ticagrelor’s antibiofilm activity and its efficacy in the treatment of *S. aureus* PJI are unknown.

**Methods:** To study the *in vitro* antibacterial and antibiofilm activity of ticagrelor, broth microdilution and crystal violet staining method were used. Ticagrelor’s effect on the expression of *S. aureus* biofilm genes (*icaA*, *icaD*, *ebps*, *fib*, *eno*, and *agr*) was studied using the relative quantification method. To test ticagrelor’s *in vivo* efficacy to treat *S. aureus* PJI, mice were randomized into five groups (*n* = 8/group): infected femoral implants treated with ticagrelor alone; infected implants treated with cefazolin alone; infected implants treated with ticagrelor and cefazolin; infected implants treated with phosphate buffer solution (PBS)-positive controls, and sterile implants-negative controls. Ticagrelor was administered orally from day 4 to day 7 post-surgery, while cefazolin was injected intravenously on day 7.

**Results:** Ticagrelor, alone and with selected antibiotics, showed *in vitro* antibacterial and antibiofilm activity against *S. aureus*. Strain-specific downregulation of biofilm-related genes, *fib*, *icaD*, *ebps*, and *eno,* was shown. In an animal model of biofilm-related *S. aureus* PJI, ticagrelor alone and combined with cefazolin significantly reduced bacterial concentrations on the implants compared with the positive control group. Ticagrelor significantly reduced bacterial dissemination to periprosthetic tissue compared with the positive controls.

**Conclusion:** Ticagrelor adjuvant therapy reduced *S. aureus* PJI in an animal model. However, this study is very preliminary to make a conclusion on the clinical implication of the findings. Based on the current results, more studies are recommended to better understand its implication.

## Background

Arthroplasty is one of the most commonly performed orthopedic procedures. However, 2.0–2.4% of these life-enhancing surgeries fail because of biofilm-related bacterial infections that are difficult to treat (Kurtz et al., 2012). *Staphylococcus aureus*, a part of normal human flora, is the most common cause of prosthetic joint infection (PJI), being involved in up to 57% of infections (Peel et al., 2012). Bio-inert medical implants coated with host proteins, such as fibrinogen, provide a rich environment for *S. aureus* attachment and biofilm proliferation (Herrmann et al., 1988). Consequently, a very low number of bacteria (<50 CFU) is enough to establish joint infection in the presence of a prosthesis compared with 10^4^ CFU in its absence (Southwood et al., 1985). Physical barriers and the presence of metabolically inert cells in the biofilm make its eradication through antibacterial therapy alone difficult (Bigger, 1944; Hoyle et al., 1992). As a result, surgical intervention to replace a prosthesis or debridement, followed by long term antibiotic therapy is the current treatment of choice (Tande and Patel, 2014). However, these procedures are traumatic and expensive with failure rates of up to 25% (Davis, 2016).

Ticagrelor is a P2Y12 receptor inhibitor antiplatelet drug used to prevent cardiac stent clotting (Springthorpe et al., 2007). In a post hoc analysis of large cardiovascular disease prevention studies, acute coronary syndrome and pneumonia patients treated with ticagrelor had a lower risk of infection-related death and showed improved lung function (Wallentin et al., 2009; Storey et al., 2014; Sexton et al., 2018). More specifically, ticagrelor protected acute coronary syndrome patients from infections by Gram-positive bacteria such as *S. aureus* (Lupu et al., 2020; Lee et al., 2021; Butt et al., 2022). This drug has also been shown to inhibit biofilm-related *S. aureus* infection in a subcutaneous prosthesis infection animal model (Lancellotti et al., 2019). Ticagrelor has also shown synergistic effects with antibiotics, rifampicin, ciprofloxacin, and vancomycin, for the *in vitro* inhibition of methicillin-resistant *S. aureus* (MRSA) (Lancellotti et al., 2019).

Ticagrelor’s molecular mechanism of *S. aureus* biofilm inhibition and its efficacy to treat *S. aureus* PJI have not been defined until now. We tested the *in vitro* antibacterial and antibiofilm activity of ticagrelor, alone and with antibiotics, and its effect on biofilm-related gene regulation. We also studied the efficacy of ticagrelor in the treatment of biofilm-related *S. aureus* infection in a PJI mouse model. We reasoned that early reintroduction of ticagrelor post-operatively may improve arthroplasty outcomes by preventing PJI.

## Materials and Methods

Two *S. aureus* clinical strains, TUH_MSSA_01 (methicillin-susceptible) and TUH_MRSA_02 (methicillin-resistant), isolated from patients treated at the Townsville University Hospital, Queensland, Australia, were used in this study. These strains were chosen from among 19 different *S. aureus* strains including an ATCC 25923 available for use in this study. The two *S. aureus* strains were chosen because they produced the most luxuriant biofilms as measured by [optical density (OD) > 4 × (negative control’s mean OD + 3 standard deviation)] (Stepanović et al., 2000). Biofilm production was induced in the *S. aureus* strains by culturing in Luria-Bertani (LB) broth at 37°C for 48 h without shaking, followed by sub-culturing in 0.5% glucose-containing LB (GLB) broth for 24 h.

### *In Vitro* Antibacterial and Antibiofilm Activity of Ticagrelor

Fifty µl of bacterial broth containing 10^5^ CFU was added to microtiter plate wells containing eight serially double-diluted ticagrelor concentrations to make the final volume 100 µL and the final ticagrelor (Sigma-Aldrich) concentrations 50 μg/ml to 0.75 μg/ml followed by incubation for 24 h at 37°C. Antibacterial activity was measured by determining the OD at 600 nm. The minimum bactericidal concentration (MBC) was determined by quantifying bacteria from wells with no visible growth, using the drop dilution method (Naghili et al., 2013). The minimum ticagrelor concentration that reduced bacterial concentration by more than 99.9% was taken as MBC. To determine the antibiofilm activity of ticagrelor, we used previously reported biofilm assay procedures with a slight modification (Singh et al., 2019). The culture supernatant was discarded and the residual biofilm was fixed with 2% sodium acetate for 10 min. Then the biofilm was stained overnight with crystal violet followed by rinsing with tap water and air drying. The crystal violet retained was then reconstituted with absolute ethanol, and OD values were measured at 570 nm. The experiments were performed in triplicates. *S. aureus* growth in ticagrelor diluent dimethylformamide (DMF) (4.15%) was used as a positive control while the sterile DMF was used as a negative control.

### *In Vitro* Combined Antibacterial and Antibiofilm Effect of Ticagrelor and Antibiotics (Cefazolin, Rifampicin, and Vancomycin)

The combined effect of ticagrelor (50 μg/ml to 0.8 μg/ml), with cefazolin (0.5 μg/ml to 0.007 μg/ml), vancomycin (2.5 μg/ml to 0.03 μg/ml), and rifampicin (0.015 μg/ml to 0.0002 μg/ml) was tested as described previously except that the final volume used was 150 µL (50 µL each of ticagrelor, antibiotic, and bacterial suspension). The fractional inhibitory concentration (FIC) index value was determined by performing the checkerboard assay (Meletiadis et al., 2010; Alhashimi et al., 2019). The combined effects of sub-inhibitory concentrations of ticagrelor and antibiotics compared with those of each alone were also tested.

## Analysis of the Effect of Ticagrelor Treatment on *S. aureus* Biofilm-Related Gene Expression

### Polymerase Chain Reaction

The ethanol precipitation method was used to extract genomic DNA (Green and Sambrook, 2017). Primers for *icaA*, *icaD*, *eno*, *fib*, *ebps*, and *agr* genes (Sigma-Aldrich) were used to detect the *S. aureus* biofilm-related genes (Table 1). A Qiagen Multiplex PCR plus kit (Qiagen, Hilden, Germany) was used for the polymerase chain reaction (PCR). The PCR reaction volume used was 10 µL and contained 0.2 µL of genomic DNA template, 1 × PCR master mix, and 200 nM of each primer. PCR parameters used were: initial denaturation (95°C, 5 min), followed by 35 cycles of denaturation (95°C, 30 s), annealing (56°C, 1.5 min), elongation (72°C, 30 s), and final extension (68°C, 10 min). PCR products were analyzed by gel electrophoresis.

**TABLE 1:** Primers used for PCR and qRTPCR.

	Oligonucleotide sequence (5′→3′)	Product size (bp)	References
PCR primers
*icaA* (F)	ACA​CTT​GCT​GGC​GCA​GTC​AA	188	Rohde et al. (2001)
*icaA* (R)	TCT​GGA​ACC​AAC​ATC​CAA​CA		
*icaD* (F)	ATG​GTC​AAG​CCC​AGA​CAG​AG	198	Rohde et al. (2001)
*icaD* (R)	AGT​ATT​TTC​AAT​GTT​TAA​AGC​AA		
*eno* (F)	ACGTGCAGCAGCTGACT	301	Tristan et al. (2003)
*eno* (R)	CAA​CAG​CAT​TCT​TCA​GTA​CCT​TC		
*ebps* (F)	CAT​CCA​GAA​CCA​ATC​GAA​GAC	180	Tristan et al. (2003)
*ebps* (R)	CTT​AAC​AGT​TAC​ATC​ATC​ATG​TTT​ATC​TTT​G		
*fib* (F)	CTA​CAA​CTA​CAA​TTG​CCG​TCA​ACA​G	405	Tristan et al. (2003)
*fib* (R)	GCT​CTT​GTA​AGA​CCA​TTT​TCT​TCA​C		
*agr* (F)	AAT​TTG​TTC​ACT​GTG​TCG​ATA​AT	135	Ferreira et al. (2013)
*agr* (R)	TGG​AAA​ATA​GTT​GAT​GAG​TTG​TT		
qRTPCR primers		Function of the related genes (Hartford et al., 2001; Downer et al., 2002; Tristan et al., 2003; Carneiro et al., 2004; Arciola et al., 2006)	
*icaA* (F)	CAA​TAC​TAT​TTC​GGG​TGT​CTT​CAC​TCT	Slime production	Kot et al. (2018)
*icaA* (R)	CAA​GAA​ACT​GCA​ATA​TCT​TCG​GTA​ATC​AT		
*icaD* (F)	TCA​AGC​CCA​GAC​AGA​GGG​AAT​A	Slime production	Kot et al. (2018)
*icaD* (R)	ACA​CGA​TAT​AGC​GAT​AAG​TGC​TGT​TT		
*eno* (F)	AAA​CTG​CCG​TAG​GTG​ACG​AA	Encode cell surface associated proteins	Kot et al. (2018)
*eno* (R)	TGT​TTC​AAC​AGC​ATC​TTC​AGT​ACC​TT		
*ebps* (F)	ACA​TTC​AAA​TGA​CGC​TCA​AAA​CAA​AAG​T	Encode cell surface associated proteins	Kot et al. (2018)
*ebps* (R)	CTT​ATC​TTG​AGA​CGC​TTT​ATC​CTC​AGT		
*fib* (F)	GAA​TAT​GGT​GCA​CGT​CCA​CAA​TT	Encode cell surface associated proteins	Kot et al. (2018)
*fib* (R)	AAG​ATT​TTG​AGC​TTG​AAT​CAA​TTT​TTG​TTC​TTT​TT		
*agr* (F)	AAT​TTG​TTC​ACT​GTG​TCG​ATA​AT	Biofilm dispersal	Ferreira et al. (2013)
*agr* (R)	TGG​AAA​ATA​GTT​GAT​GAG​TTG​TT		
*rpoB* (F)	CAG​CTG​ACG​AAG​AAG​ATA​GCT​ATG​T		Kot et al. (2018)
*rpoB* (R)	ACT​TCA​TCA​TCC​ATG​AAA​CGA​CCA​T		
*gmk* (F)	CCA​TCT​GGA​GTA​GGT​AAA​GG		Theis et al. (2007)
*gmk* (R)	CTACGCCATCAACTTCAC		

### RNA Extraction for Quantitative Reverse Transcriptase Polymerase Chain Reaction

RNA was extracted from 8 h *S. aureus* test and positive control cultures treated with 12.5 μg/ml ticagrelor and 1% DMF, using a Qiagen RNeasy mini kit (Qiagen, Hilden, Germany). Ticagrelor 12.5 μg/ml showed significant antibiofilm activity without inhibiting planktonic growth. A Nanodrop 2000C spectrophotometer (Thermo Fisher Scientific, United States) was used to measure the RNA quality and quantity.

### Measurement of Gene Expression

A Bio-Rad iTaq universal SYBR green one-step kit (Bio-Rad, United States) was used for qRTPCR. The effect of ticagrelor on the expression of biofilm-related *S. aureus* genes, *icaA*, *icaD*, *eno*, *fib*, *ebps*, and *agr*, was tested in triplicate using relative quantification method (Table 1). The level of the effect was measured by comparative C_t_ (ΔΔC_t_) method (Livak and Schmittgen, 2001). The results were presented as fold change ± standard deviation in comparison with the positive control. Reference genes used were *rpoB* and *gmk* because their expression was treatment-independent. These genes were selected from among 16 different candidate reference genes because they were most stably expressed in the experimental condition used. The qRTPCR was carried out in 10 µL volume that contained 5 µL 2 × iTaq universal SYBR green reaction mix, 0.125 µL iScript reverse transcriptase, 0.8 ng RNA template in 1 µL volume, 1 nM of primer mix in 1 µL volume, and 2.875 µL of nuclease-free water. The thermo-cycler parameters used were: reverse transcription (50°C, 10 min), polymerase activation and DNA denaturation (95°C, 1 min), 40 cycles of denaturation at 95°C for 10 s, and annealing/extension + plate read at 60°C for 30 s.

### Animal Study

Ethical approval to conduct the animal study was granted by the James Cook University Animal Ethics Committee (AEC2486). Six to ten week-old C57BL/6 female mice (Animal Resources Centre, Western Australia) were randomized into five groups (*n* = 8/group): 1) infected implants treated with ticagrelor alone; 2) infected implants treated with cefazolin alone; 3) infected implants treated with ticagrelor and cefazolin; 4) infected implants treated with phosphate buffer solution (PBS) (positive control); and 5) sterile implants (negative control).

### Surgical Technique

The animal model used to emulate prosthesis-related joint infection was described by Bernthal et al. (2010). Buprenorphine (0.2 mg/kg, sc) was administered 30 min pre-surgery, while ketamine/xylazine (90 mg/kg/10 mg/kg, ip) was used just before surgery. Hair was removed from the right thigh and the skin was disinfected with povidone-iodine. An incision was made above the right knee to displace the knee cap and to access the femoral intercondylar notch. Then the femoral intramedullary canal was reamed manually with a 26 G needle and an orthopedic-grade stainless steel Kirschner (K)-wire (diameter 0.6 mm) was inserted to leave its 1 mm cut end protruding into the joint space. The K-wire was contaminated with 500 CFU of *S. aureus* in 2 µL PBS bacterial suspension pipetted into the joint space. The knee cap was replaced and the skin was closed with a 5-0 absorbable suture. Combined subcutaneous (0.2 mg/kg) and oral (2.5 ml/160 ml drinking water) buprenorphine was given as an analgesic for 72 h.

### Treatment Administered

The ticagrelor alone treatment group was treated with ticagrelor (3 mg/kg loading dose followed by 1.5 mg/kg twice daily in 100 µL volume) orally from day 4 to day 7 post-surgery. This is the dose/weight equivalent to human treatment (Lancellotti et al., 2019). This drug intervention timing was used because it mimics the time of reintroduction of the antiplatelet drug in human arthroplasty surgery to avoid drug-related bleeding from fresh wounds. Similarly, the cefazolin alone treatment group was injected with a single intravenous dose of cefazolin (2.5 mg/kg) on day 7 post-surgery. To test whether the biofilm dispersed by ticagrelor would be killed by an antibiotic, the ticagrelor plus cefazolin treatment group was administered ticagrelor from day 4 to day 7 followed by a single cefazolin (2.5 mg/kg) dose on day 7 post-surgery. The cefazolin dose given was not intended to eradicate the biofilm infection, rather it was designed to measure whether any combined effect was present with ticagrelor. Clinical parameters such as weight, eating, drinking, mobility, and pain indicators were recorded daily. The mice were kept alive for sufficient time after the ticagrelor and cefazolin treatments ended to let the infection develop again if not eradicated. On day 14 post-surgery, the mice were culled using carbon dioxide, and implants and surrounding tissues were collected for bacteriological analysis.

### Bacterial Culture of K-Wires and Tissues

The extracted K-wires collected were rinsed with sterile cold PBS to wash off planktonic cells. The K-wires were then sonicated at 44 khz in 5 ml cold LB for 5 min using a water bath sonicator to disrupt biofilm and remove the attached cells. Periprosthetic tissues were collected in 800 µL ice-cold PBS to slow down bacterial multiplication, and then homogenized using a Navy Lysis Kit (BioTools, Australia). The sonication fluids and the homogenized tissues were serially ten-fold diluted and cultured in LB and Mannitol Salt Agar (MSA) for 48 h at 37°C. Bacterial colony counts were presented as log CFU/ml.

### Statistical Analysis

Graphpad version 8.2.0 (GraphPad Software, San Diego, California, United States) was used for performing one-way ANOVA followed by the Tukey multiple comparison test. *p*-Value <0.05 was taken as statistically significant.

## Results

### Antibacterial and Antibiofilm Activity of Ticagrelor

**FIGURE 1:**
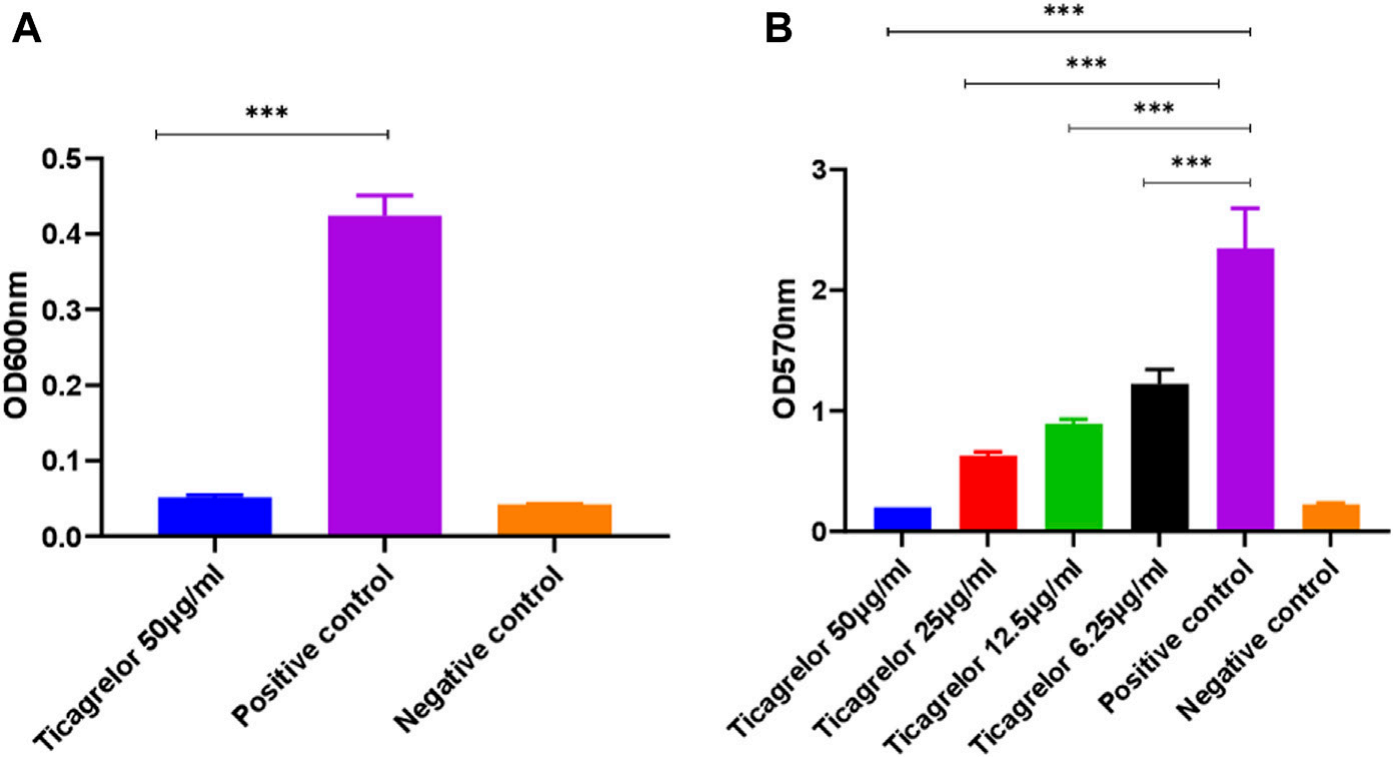
*S. aureus* planktonic **(A)** and biofilm **(B)** growth in the presence of ticagrelor. Experiments were performed in triplicate (N = 3) and data were presented as mean ± standard deviation (SD) with the error bars indicating SD (***< 0.001). Ticagrelor showed antibacterial and antibiofilm activity against *S. aureus*.

Ticagrelor did not show strain-specific activity judged by identical results for experiments using the TUH_MSSA_01 and TUH_MRSA_02 isolates. The minimum bactericidal concentration (MBC) of ticagrelor for both strains was 50 μg/ml. Ticagrelor also exhibited significant antibiofilm activity (Figure 1).

### Antibacterial and Antibiofilm Activity of Ticagrelor in Combination With Antibiotics

**FIGURE 2:**
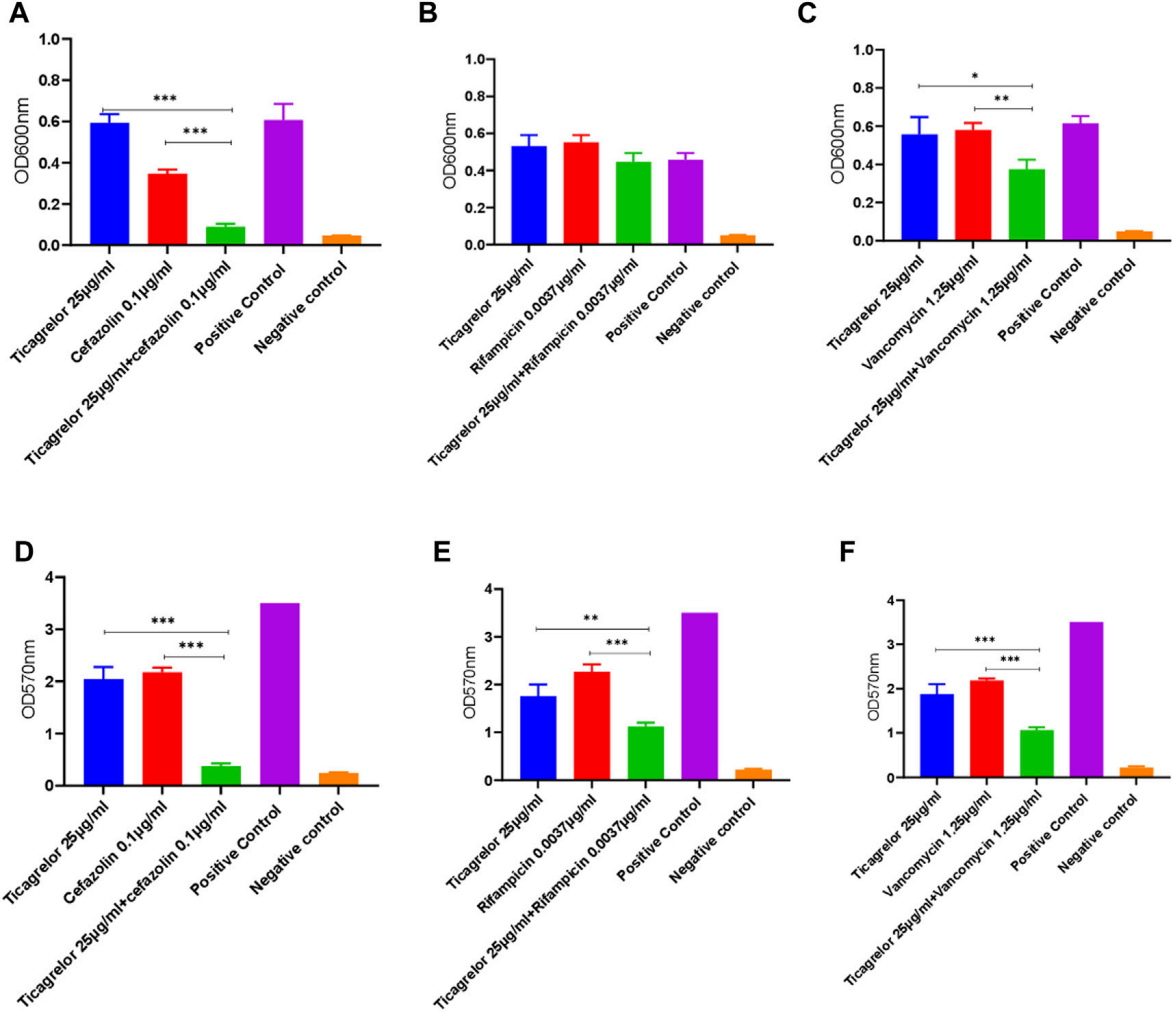
Combined antibacterial **(A,B,C)** and antibiofilm **(D,E,F)** activity of sub-inhibitory concentrations of ticagrelor and antibiotics (cefazolin, rifampicin, and vancomycin) in comparison with each alone. A sub-inhibitory ticagrelor concentration was used in this experiment. Ticagrelor in combination with antibiotics had higher activity than ticagrelor alone. Data are presented as mean (N = 3) ± standard deviation (SD) and error bars indicate SD (*** = *p* < 0.001, ** = <0.01, and * = <0.05).

Different ticagrelor and antibiotic (cefazolin, rifampicin, and vancomycin) concentrations were tested for their combined effect on the planktonic and biofilm growth of *S. aureus*. TUH_MRSA_02 being resistant to cefazolin, ticagrelor and cefazolin combination was not tested in this strain. For the antibiotic and ticagrelor combinations tested in both strains, there was no strain-specific difference. Calculated fractional inhibitory concentration (FIC) index values showed that additive antibacterial and antibiofilm activity was present for ticagrelor in combination with cefazolin and vancomycin (Figure 2). No additive antibacterial but antibiofilm effect was shown with ticagrelor and rifampicin combination. The sub-inhibitory concentrations of ticagrelor and the antibiotics alone shown in Figure 2 had either no or minimal antibacterial and antibiofilm activity.

### Effect of Ticagrelor Treatment on Biofilm-Related *S. aureus* Genes Expression

**FIGURE 3:**
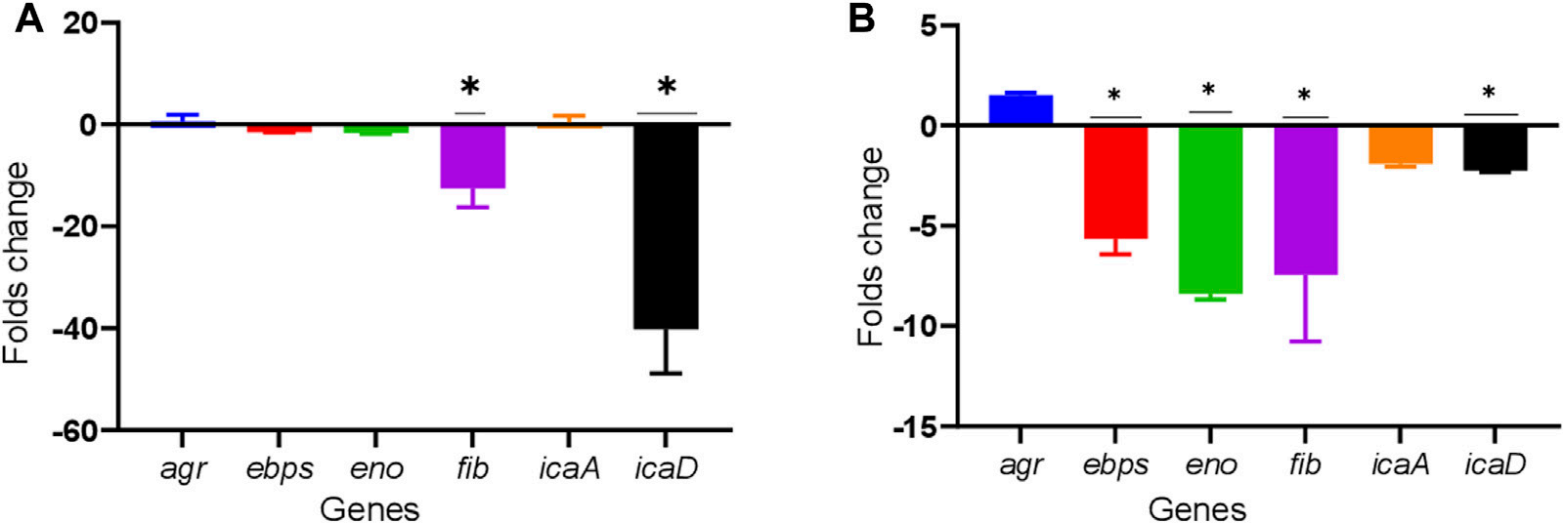
Downregulation of biofilm-related genes in TUH_MSSA_01 **(A)** and TUH_MRSA_02 **(B)** strains after 8 h of growth in the presence of ticagrelor (12.5 ug/ml). The reference genes used were *gmk* and *rpoB*. The effect of ticagrelor treatment on gene expression was determined by the comparative C_t_ (ΔΔC_t_) method. Data are presented as mean fold changes (N = 3) ± standard deviation (SD) compared with ticagrelor diluent (1% dimethylformamide)-treated control and error bars indicate SD (* = down regulated by > 2 folds).

All the biofilm-related genes tested, *icaA*, *icaD*, *eno*, *fib*, *ebps*, and *agr*, were detected in both the TUH_MSSA_01 and TUH_MRSA_02 strains. Ticagrelor showed strain-specific downregulation of these biofilm-related genes. Genes *fib* and *icaD* were downregulated in TUH_MSSA_01 while *ebps*, *eno*, *fib*, and *icaD* were downregulated in TUH_MRSA_02 (Figure 3).

### Effect of Ticagrelor Treatment Alone and With Cefazolin on Bacterial Concentration on K-Wire Implants and Periprosthetic Joint Tissues

**FIGURE 4:**
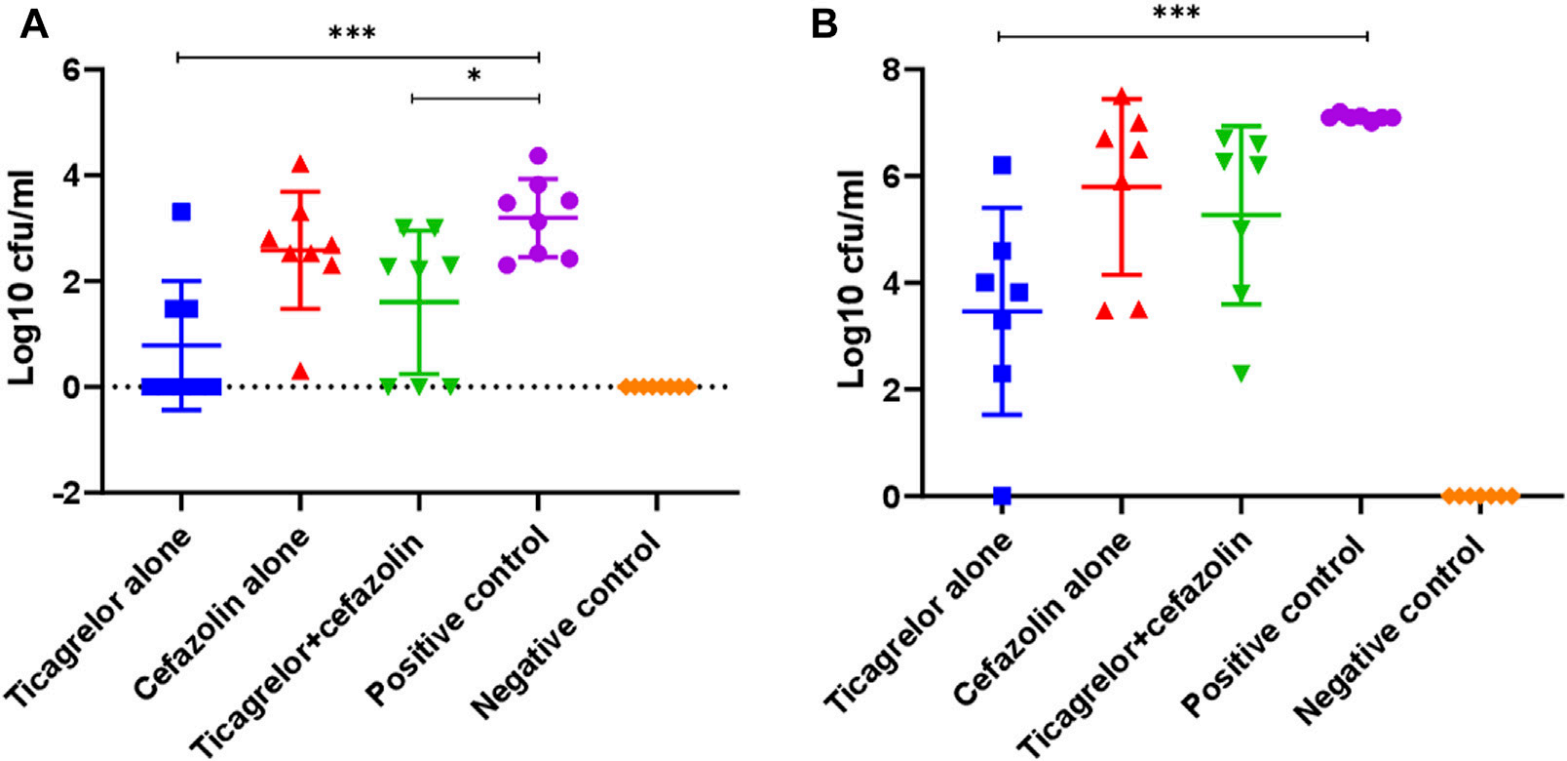
Bacterial concentration on K-wire **(A)** and peri-prosthetic tissue **(B)** from different treatment and control groups on day 14 post-surgery (***p < 0.001, *p < 0.05). Ticagrelor alone reduced bacterial concentration in both implant and periprosthetic tissue, while ticagrelor and cefazolin combination reduced bacterial concentration only on implants. The data are presented as mean log10 CFU/ml ± standard deviation (SD).

We proceeded to use cefazolin in the animal study because this is the most commonly used antibiotic in arthroplasty and showed better *in vitro* combined effect with ticagrelor than the other antibiotics tested. We tested the effect of ticagrelor, alone and in combination with cefazolin, on the TUH_MSSA_01-infected K-wire implants and periprosthetic tissues in a mouse model. Ticagrelor alone and in combination with cefazolin significantly reduced bacterial concentration on implants extracted from experimentally infected mice knees compared with the PBS-treated control (log10cfu/ml, 0.8 versus 3.2, *p* < 0.001, and 1.6 versus 3.2, *p* < 0.05) (Figure 4). Ticagrelor reduced bacterial dissemination in the periprosthetic tissues compared with the positive control (log10 cfu/ml, 3.6 versus 7.1, *p* < 0.001). There was a non-significant increase in bacterial concentrations in implants and periprosthetic tissues from mice administered cefazolin in addition to ticagrelor compared with that from mice administered ticagrelor alone. However, when compared with the PBS-treated positive control, the inhibitory activity of ticagrelor alone was statistically more significant with a *p*-value <0.001 than that of ticagrelor plus cefazolin where a *p*-value was <0.05.

## Discussion

Ticagrelor, an antiplatelet drug, shows *in vivo* and *in vitro* antibacterial and antibiofilm activity against *S. aureus* (Lancellotti et al., 2019). To our knowledge, the use of ticagrelor for the treatment of prosthesis-related *S. aureus* joint infection and the underlying molecular mechanisms of its antibiofilm activity have not been investigated. We studied the efficacy of ticagrelor used as non-antimicrobial adjuvant therapy to treat biofilm-related *S. aureus* infection in a prosthetic joint infection (PJI) mouse model and the associated molecular mechanism.

Ticagrelor showed *in vivo* antibacterial and antibiofilm activity such that it reduced PJI due to the TUH_MSSA_01 strain in an animal model. The antibiofilm activity is attributed to the inhibition of critical biofilm-related genes. Ticagrelor also exhibited *in vitro S. aureus* planktonic and biofilm growth inhibition, and additive effects with the antibiotics cefazolin, and vancomycin. However, ticagrelor showed enhanced antibiofilm activity but no additive antibacterial effect when combined with rifampicin.

Only one previous study that used a pre-contaminated subcutaneous foreign body *S. aureus* infection mouse model has reported the *in vitro* and *in vivo* antibacterial and antibiofilm activity of ticagrelor against *S. aureus,* including MRSA (Lancellotti et al., 2019). Mice were subcutaneously implanted with polyurethane disks contaminated with *S. aureus*, and bioluminescent imaging was performed to determine the efficacy of ticagrelor treatment (Lancellotti et al., 2019). While both the previous and our study reported ticagrelor’s *in vivo* and *in vitro* antibacterial and antibiofilm activity including the enhanced activities of antibiotics, there were some key differences. The magnitude of antibacterial activity shown was higher in the previous study (minimum bactericidal concentration = 20 μg/ml) (Lancellotti et al., 2019) and we did not report enhanced antibacterial activity of rifampicin. The discrepancy in the results on ticagrelor’s antibacterial activity between the previous and our study might be due to different bacterial strains or the methods used. For instance, we used broth micro-dilution method followed by the drop dilution method for a viable count, while the former study used a time-kill assay and disk diffusion assay (Lancellotti et al., 2019). Another study has reported the minimum inhibitory concentration of ticagrelor to be 33 μg/ml and its *in vitro* additive effect with cefazolin and ertapenem against methicillin-susceptible *S. aureus* (MSSA) (Ulloa et al., 2021).

For the first time, we have explored the genetic mechanism of the antibiofilm effect of ticagrelor in both TUH_MSSA_01 and TUH_MRSA_02 strains. We have demonstrated the strain-specific downregulation of some key biofilm-related genes: *fib, icaD*, *ebps*, and *eno*. In general, biofilm formation involves quorum sensing. Consequently, biofilm inhibition involves the combination of the lowering of bacterial concentration and the regulation of different biofilm-related genes. Genes *eno*, *ebps*, and *fib* initiate biofilm formation through the expression of cell wall-associated proteins that promote *S. aureus* attachment and colonization (Hartford et al., 2001; Downer et al., 2002; Tristan et al., 2003; Carneiro et al., 2004); *icaA* and *icaD* produce slime and help in biofilm maturation (Arciola et al., 2006). So, the downregulation of all or any one of these genes affects biofilm production negatively. Strain-specific expressions of different biofilm-related genes in weak and strong biofilm producer *S. aureus* have already been reported (Kot et al., 2018). Although both the strains we used were strong biofilm producers, TUH_MSSA_01 produced a more luxuriant biofilm than TUH_MRSA_02. The *agr* gene helps in *S. aureus* biofilm dispersal (Vuong et al., 2000). However, amyloid fiber, a product of *agr* quorum sensing, is known to stabilize the biofilm (Schwartz et al., 2012). So, the role of *agr* in *S. aureus* biofilm may be strain-specific (Vuong et al., 2000), and we did not notice any effect of ticagrelor treatment on this gene.

Since TUH_MRSA_02 is cefazolin-resistant, we proceeded to test the efficacy of ticagrelor, alone and in combination with cefazolin, in an animal model using TUH_MSSA_01 only. In addition, PJI is more frequently caused by MSSA than MRSA (Manning et al., 2020). Ticagrelor demonstrated *in vivo* antibacterial and antibiofilm activity against the TUH_01_MSSA strain used. In the animal prosthetic joint infection model, it reduced bacterial concentration on the K-wire and periprosthetic tissue. However, none of the treatments used in this study sterilized the infection. In a clinical context, for a successful cure of a PJI it is necessary to sterilize the infection. While the cefazolin dose we used in this study was sub-optimal, the standard regimen for prophylactic cefazolin in arthroplasty is a single 2 g dose administered intravenously pre-surgery (Arthroplasty society of Australia, 2018). Since ticagrelor alone showed *in vitro* sterilization of *S. aureus* growth, it might also be possible to attain this *in vivo* through the variation of drug dosing and timing. These factors could be investigated with more animal studies. Reduction in biofilm formation and bacterial dissemination to surrounding tissue due to ticagrelor treatment, with the same dosages as in our study, in a pre-contaminated subcutaneous disc *S. aureus* infection mouse model has been reported (Lancellotti et al., 2019).

When antiplatelet drugs were recommenced as early as possible after arthroplasty in patients, there was no increase in bleeding risk (Nandi et al., 2012). However, this observation may be unique to this particular study and the chances of bleeding-related complications when antiplatelet drugs are resumed immediately post-surgery still exist. To minimize bleeding risk in patients already under antiplatelet therapy, it is recommended to discontinue antiplatelet drugs 5 days before surgery and resume them 72 h post-surgery (Rahman and Latona, 2014). When ticagrelor is used before sufficient hemostasis and wound healing are achieved, this may lead to an increase in bleeding, delay in wound healing, and consequently to an increase in the chances of infection. However, the earliest possible use of ticagrelor post-surgery may prevent biofilm-related infections effectively improving outcomes for an arthroplasty surgery. As the procedure we performed emulated high-bleeding-risk orthopedic surgery, we waited for 3 days until sufficient hemostasis and wound healing were seen, and then commenced ticagrelor treatment. Consequently, we did not encounter complications associated with ticagrelor-related bleeding and ticagrelor improved the overall outcome of arthroplasty in the animal model.

Ticagrelor in combination with cefazolin has never been used before in the treatment of bacterial infections in an animal model. *In vitro,* the combination of ticagrelor and cefazolin showed greater antibacterial and antibiofilm activity than ticagrelor alone. In our study, the reverse pattern of inhibition was seen *in vivo* as ticagrelor alone showed better antibacterial and antibiofilm activity than the ticagrelor and cefazolin combination. Thus, the reduction in *S. aureus* infection seen in our PJI mouse model appears to be mainly due to ticagrelor. However, more animal studies to determine the pharmacokinetics and pharmacodynamics of ticagrelor, alone and in combination with cefazolin, mainly in relation to its antibacterial and antibiofilm activity might give better insight into the *in vivo* antagonistic effect of ticagrelor and cefazolin.

Platelets mediate *S. aureus* clearance, while *S. aureus* α-toxin induces thrombocytopenia (Ali et al., 2017; Sun et al., 2021). At a physiological concentration, ticagrelor had a protective effect on platelets against *S. aureus* α-toxin and enhanced platelet-mediated MRSA and MSSA killing (Sun et al., 2021; Ulloa et al., 2021). Given the maximum achievable systemic ticagrelor concentration (1.2 μg/ml), with standard dosages for acute coronary syndrome is significantly lower than the direct inhibitory supraphysiologic concentrations (20 μg/ml to 50 μg/ml) (Ulloa et al., 2021), the reduction in the infection seen in our study might be platelet-mediated and not related to the direct antibacterial effect of ticagrelor. Alternatively, biofilm-related *S. aureus* infection involves interactions between bacterial clumping factor A, GPIIb/IIIa platelet receptor, and fibrinogen (Siboo et al., 2001; Ditkowski et al., 2021). Platelet inactivation by ticagrelor might prevent *S. aureus* attachment to platelet and consequently to the host tissue, leading to infection clearance.

*S. aureus* does not develop resistance to ticagrelor as easily as it does to conventional antibiotics (Lancellotti et al., 2019). When either MSSA or MRSA strains were serially treated with sub-inhibitory concentrations of ofloxacin or rifampicin or ticagrelor, the development of resistance with the antibiotics was observed but not with ticagrelor (Lancellotti et al., 2019).

Ticagrelor also showed *in vitro* antibacterial and antibiofilm activity against *S. epidermidis* and vancomycin-resistant *Enterococcus* (VRE) but not against *Klebsiella pneumoniae* and *Pseudomonas aeruginosa* (data not shown). We concluded that ticagrelor has the potential to prevent biofilm formation by the commonest Gram-positive bacteria, but not Gram-negative bacteria, responsible for causing PJI. This conclusion is congruent with the findings of the previous study (Lancellotti et al., 2019). Ticagrelor’s *in vitro* anti-bacterial activity for MRSA and VRE indicates that ticagrelor’s mode of antibacterial action is not same as that of cefazolin and vancomycin.

Biofilm dispersal using adjuvant, non-antimicrobial therapy with ticagrelor may improve the success rate of PJI treatment. Further animal model and human observational data indicating a benefit of ticagrelor for the treatment of biofilm-related infections may support intervention trials in humans. Our study lays a foundation for research in this direction. Repurposing this safe Food and Drug Administration-approved drug for PJI due to *S. aureus* could be cheap and rapid.

**Limitations of the study**: We were not able to study the mechanism of antibacterial activity of ticagrelor. However, the leakage of cellular components has been identified as a possible mechanism for its bactericidal activity (Phanchana et al., 2020). The pathogenesis for PJI is complex and the animal model we used was chosen for its simplicity but it is not an ideal representation of human infections. Studies using large animal prosthetic joint infection models that use the same materials and techniques as used in modern arthroplasty could better represent human PJI pathogenesis. In our study, while ticagrelor reduced *S. aureus* prosthetic joint infection in an animal model, caution should be taken while interpreting the results. Only two clinical *S. aureus* strains were used in the *in vitro* and molecular experiments and one in the *in vivo* experiment. The *in vivo* data generated in this study are very preliminary to make a conclusion about their clinical implication. More studies using different *S. aureus* strains in different prosthetic joint infection animal models are recommended.

## Data Availability

The original contributions presented in the study are included in the article/Supplementary Material; further inquiries can be directed to the corresponding author.
